# Genomic advancement: Aiming to affirm and improve human life^☆^

**DOI:** 10.1016/j.atg.2016.11.002

**Published:** 2016-11-09

**Authors:** Carol Isaacson Barash, Nigel G. Halford

**Affiliations:** a Helix Health Advisors, US; b Rothamsted Research, UK

Challenges to improving human health and well being are both within and outside our control despite the best of scientific advances. While advances continue to offer improved technologies, and thus our ability to not only improve detection and treatment of disease, preventing the roots of illness from taking hold involves environmental and social factors. For example, climate and politics can aid or abet the best of health improvement strategies yielding untoward consequences for those in need who lack substantial control over their access to food and healthcare.

In this issue, we present a collection of articles that contribute to our understanding of genomics, ethical and policy factors necessary to improving health. We start with a special section on the genomics of plant breeding and move to translational genomics and health improvement.

## 1 Special section: crop genomics and food security

Agriculture must overcome some daunting challenges if it is to meet the demands of an increasing world population and rising per capita consumption in the coming decades, including climate change, insufficient fresh water, soil erosion, salination, and pollution, as well as the use of crop commodities for fuel as well as food, the advent of peak oil, and competition for land use. If it is to meet the demand for food in the face of these challenges, plant breeding will have to bring about increases in crop yield at a much faster rate than it is currently achieving, while improving, or at least maintaining, food quality and safety. Genomics and systems biology, along with biotechnology, will be key to making this possible.

This special section on agricultural genomics comprises three papers on wheat. Although wheat production has been overtaken by that of maize and rice in recent years, wheat grain remains the most traded crop commodity on world markets, the leading crop source of protein and, along with rice, one of the two most important crop products for human consumption. However, wheat yield has leveled off in many regions of the world in the last two to three decades; a trend that must be reversed as demand increases.

In the first paper, Carlos Guzman and colleagues review the use of genomic selection in the Global Wheat Program of the International Center for Maize and Wheat Improvement (CIMMYT) in Mexico. This program aims to increase the productivity of wheat cropping systems in developing countries, but its priorities of high grain yield, disease resistance, tolerance to abiotic stresses (drought and heat), and end-use quality are familiar to wheat breeders everywhere. Genomic selection using genome-wide single nucleotide polymorphism markers is being applied to multiple quantitative traits in a breeding pipeline involving thousands of lines.

Olufunmilayo Ladejobi and colleagues, led by Alison Bentley at the National Institute of Agricultural Botany (NIAB) in Cambridge, UK, review the use of multi-parent populations in quantitative trait mapping and elucidating the genetic basis of complex crop traits.

Lastly, Keywan Hassani-Pak of Rothamsted Research in the UK and colleagues describe bio-mathematical approaches to investigating the genetic basis of crop traits, incorporating gene discovery and the construction of networks of genetic interactions. These approaches, which the team applies to wheat and its close relative, barley, will enable researchers to mine the huge amount of genetic information that is already available but is dispersed in databases that use a variety of formats and have variable quality and coverage.

Together, these three reviews, describe quite different technologies and illustrate the potential that genomics has for accelerating crop genetic research and its application in crop breeding programs.

## 2 Special section: social considerations in translational genomics

The path to ensuring sound and ethical application of translational genomics requires arguably greater scholarly attention to social factors. In this last section, we present articles that advance our knowledge of these factors and their relevance to achieving the desired benefit of genomic applications.

The first paper by Rahimzadeh, et al. addresses the as yet unknown path from cell-based therapies and products (CTP) manufacturing to translation to clinical adoption. Filling an empirical gap in the literature, this study elucidates Canadian institutional practices and stakeholder perceptions in efforts to advance knowledge about how best to ensure appropriate and ethical commercialization and regulation.

Reznichenko, et al. discuss technical and ethical challenges in the clinical use of mitochondrial transfer to prevent mitochondrial disease; success rates of various techniques, counter arguments against potential mitochondrial-nuclear genome incompatibility and various clinical applications.

Lastly, Aarden, et al. address the need for scholarly attention to social infrastructures into which genomic interventions may be delivered to patient populations. The paper explores how the assessment of familial breast cancer risks was ‘translated’ into routine health care in Germany, the Netherlands and the UK, evincing why translation to the clinic is an area of social, as well as technical, concern requiring collective decision-making.

Finally, with regret, the final issue of 2016 is *the* final issue of ATG/Elsevier. It has been my personal honor to work with such an esteemed editorial board and my hope that we will continue our work together in a different context. I hope that you, our readers, have benefited and enjoyed our issues over the past three years.

